# Mogrol Derived from *Siraitia grosvenorii* Mogrosides Suppresses 3T3-L1 Adipocyte Differentiation by Reducing cAMP-Response Element-Binding Protein Phosphorylation and Increasing AMP-Activated Protein Kinase Phosphorylation

**DOI:** 10.1371/journal.pone.0162252

**Published:** 2016-09-01

**Authors:** Naoki Harada, Mikako Ishihara, Hiroko Horiuchi, Yuta Ito, Hiromitsu Tabata, Yasushi A. Suzuki, Yoshihisa Nakano, Ryoichi Yamaji, Hiroshi Inui

**Affiliations:** 1 Division of Applied Life Sciences, Graduate School of Life and Environmental Sciences, Osaka Prefecture University, Sakai, Osaka, Japan; 2 Biochemical Laboratory, Saraya Company, Ltd., Kashiwara, Osaka, Japan; 3 Center for Research and Development of Bioresources, Osaka Prefecture University, Sakai, Osaka, Japan; 4 Department of Nutrition, Osaka Prefecture University, Habikino, Osaka, Japan; Kobe University, JAPAN

## Abstract

This study investigated the effects of mogrol, an aglycone of mogrosides from *Siraitia grosvenorii*, on adipogenesis in 3T3-L1 preadipocytes. Mogrol, but not mogrosides, suppressed triglyceride accumulation by affecting early (days 0–2) and late (days 4–8), but not middle (days 2–4), differentiation stages. At the late stage, mogrol increased AMP-activated protein kinase (AMPK) phosphorylation and reduced glycerol-3-phosphate dehydrogenase activity. At the early stage, mogrol promoted AMPK phosphorylation, inhibited the induction of CCAAT/enhancer-binding protein β (C/EBPβ; a master regulator of adipogenesis), and reduced 3T3-L1 cell contents (*e*.*g*., clonal expansion). In addition, mogrol, but not the AMPK activator AICAR, suppressed the phosphorylation and activity of the cAMP response element-binding protein (CREB), which regulates C/EBPβ expression. These results indicated that mogrol suppressed adipogenesis by reducing CREB activation in the initial stage of cell differentiation and by activating AMPK signaling in both the early and late stages of this process.

## Introduction

The increase in the number of people with obesity, which is associated with excess fat accumulation, is a major global health problem [1–3]. Abdominal visceral fat mass is a key feature of metabolic syndrome, which is associated with an increased risk for cardiovascular disease and mortality [1–3]. Imbalanced energy intake and expenditure result in an accumulation of body fat within adipocytes, which primarily store fuel as triglyceride (TG). The differentiation of preadipocytes to adipocytes is important for regulating fat mass. 3T3-L1 cells represent a well-characterized model of adipocyte differentiation [4, 5]. Adipogenesis is categorized into early, middle, and late stages [4]. In the early stage, CCAAT/enhancer-binding protein β (C/EBPβ) plays an essential role in adipocyte differentiation, and ectopic expression of C/EBPβ is sufficient to induce adipogenesis in 3T3-L1 cells [4–6]. C/EBPβ induces mitotic clonal expansion, associated with approximately two rounds of cell division in relation to initiation of adipogenesis [7]. The expression of C/EBPβ is regulated by cAMP response element-binding protein (CREB) [8]. The accumulation of lipids is initiated at the middle stage and is accelerated in the late stage of differentiation [6]. The activity of glycerol-3-phosphate dehydrogenase (GPDH), which produces glycerol 3-phosphate from dihydroxyacetone phosphate, is used as a marker of adipocyte differentiation that is independent of TG accumulation [5, 9].

AMP-activated protein kinase (AMPK) acts as a sensor for cellular energy states and regulates cellular energy metabolism [10]. In adipocyte differentiation, the activation of AMPK by phosphorylation suppresses early-stage clonal expansion and switches from ATP-consuming processes, such as lipid synthesis, to ATP production; this inhibits lipid accumulation at the late stage of differentiation [11–14]. Expression of constitutively active and dominant negative forms of AMPK, as well as the generation of AMPKα1 knockout animals, have revealed its role in suppressing the accumulation of TG [15].

*Siraitia grosvenorii* Swingle, a traditional Chinese medicinal plant, has particular sweetness properties and is used as a noncaloric sugar substitute. The compounds responsible for its sweet taste have been identified as the triterpene glycosides known as mogrosides [16]. Mogrosides I to V are named after the number of conjugated glucose molecules, and mogroside V is the most abundant form present in *S*. *grosvenorii* [17]. Tri- to penta-glucose-conjugated mogrosides are several dozens to several hundred-fold sweeter than sucrose [18]. Mogrol is an aglycone of mogrosides and is the major form absorbed into the body following ingestion of mogrosides [17]. Mogrol is produced from mogrosides in the digestive tract by acid hydrolysis, digestive enzymes, or the actions of intestinal microflora [17].

The usage of functional foods provides a promising strategy for the prevention of obesity [19]. Several phytochemicals such as catechins [20], flavonoids [21], and carotenoids [22] produce beneficial effects by inhibiting lipid accumulation in adipocytes. Mogrosides possess anti-diabetic activities [23, 24] and mogrol was shown to increase AMPK activity in hepatocytes [25]. However, its effect on adipocytes has not yet been established. In the present study, we demonstrated that mogrol suppressed adipocyte differentiation in 3T3-L1 cells via at least two different mechanisms.

## Materials and Methods

### Reagents

The triterpene glycoside extract (30% Mogroside V), prepared from fresh fruit of *S*. *grosvenorii*, was purchased from the Guilin S&T New Tech Co. (Guilin, China). Mogroside V and mogroside IVE were purified from this triperpene glycoside extract by column chromatography. Mogroside IIIE, mogroside IIE, mogroside IE and mogrol were prepared by the enzyme or acid hydrolysis of mogroside V [17, 26]. These compounds were purified at least 96% and dissolved in DMSO. In a cell-based assay, final DMSO concentrations were less than 0.1%.

### Cell culture

Murine preadipocyte 3T3-L1 cells were obtained from the Japanese Collection of Research Bioresources (Osaka, Japan). Cells were maintained in Dulbecco’s modified Eagle’s medium supplemented with 4.5 g/L glucose, 10% bovine serum (16170–078; Life Technologies, Carlsbad, CA, USA), and antibiotics (100 U/mL penicillin G and 100 μg/mL streptomycin sulfate). 3T3-L1 preadipocytes were differentiated to adipocytes using a routine procedure [27]. Briefly, at 2 days post-confluency (day 0), the medium was exchanged to differentiation medium containing 4.5 g/L glucose, 10% fetal bovine serum, and the above antibiotics, and the cells were stimulated with 10 μg/mL insulin, 1 μM dexamethasone (DEX), and 0.5 mM 3-isobutyl-1-methylxanthine (IBMX). The medium was replaced by fresh differentiation medium containing 10 μg/mL insulin every 2 days, and the cells were harvested on day 8. Control cells were not exposed to differentiation stimuli and harvested on day 8. Cells were pretreated with mogrol for 30 min before stimulation with the inducers (on day 0) and treated with mogrol at every exchange of medium (on days 2, 4, and 6). Cells were maintained at 37°C in a 5% CO_2_/95% air atmosphere with 98% humidity.

### Oil red O staining

Cells were washed twice with phosphate-buffered saline (PBS; 137 mM NaCl, 2.68 mM KCl, 8.1 mM Na_2_HPO_4_, 1.47 mM KH_2_PO_4_) and fixed in 10% formaldehyde for 10 min. After washing twice with PBS, cells were incubated in 60% isopropanol for 1 min, followed by further incubation with Oil Red O stain solution (60% isopropanol containing 0.18% Oil Red O). Cells were washed once with 60% isopropanol and twice with PBS prior to analysis using microscopy (BZ9000, Keyence, Osaka, Japan).

### Measurement of cellular levels of TG and DNA, and GPDH activity

3T3-L1 cells that had been differentiated on 6-well plates were harvested on day 8 after washing twice with PBS. Lipids were extracted by the classical Folch method [28], with minor modifications and TG levels were measured using the L-type TG-M kit (Wako Pure Chemical Industries, Osaka, Japan) with triolein as a standard. For measurement of the cellular DNA level, cells were resuspended in PBS and incubated with Hoechst 33342 for 10 min. Calf thymus DNA (Nacalai Tesque, Kyoto, Japan) was used as the standard. Fluorescence intensity was measured using a Fluoroscan Ascent FL microplate reader (Labsystems, Helsinki, Finland) at an excitation wavelength of 355 nm and an emission wavelength of 460 nm. For measurement of GPDH activity, cells were sonicated in 100 μL of cell lysis buffer (50 mM Tris-HCl, pH7.5, 150 mM NaCl, 0.5% Nonidet P-40, 2 mM EDTA, 10 mM sodium pyrophosphate, 0.1 mM DTT, 1 mM phenylmethylsulfonyl fluoride, 10 μg/mL leupeptin, and 1 μg/mL aprotinin). After centrifugation at 20,000 × g for 10 min, 2-mercaptoethanol was added to the supernatant to achieve a final concentration of 1 mM and this was used as a crude cell extract. GPDH activities were measured as described previously [29] with minor modification. Briefly, the cell lysate (20 μL) was mixed with 980 μL of the assay mixture (100 mM Tris-HCl, pH7.5, 2.5 mM EDTA, 0.1 mM 2-mercaptoethanol, 0.12 mM NADH, 0.2 mM dihydroxyacetone phosphate) and incubated at 25°C. The change in absorbance at 340 nm was measured using a spectrophotometer (V550, JASCO, Tokyo, Japan). One unit of GPDH activity was defined as consuming 1 nmol NADH per min in a 1 mL reaction mixture (light path = 1 cm).

### Cell Viability Assay

3T3-L1 cells were plated onto a 48-well plate. After confluence, cells were incubated in the presence of mogrol for 8 days. Medium with mogrol was replaced to fresh medium every 2 days. The cells were then incubated in fresh medium containing 5% AlamarBlue (Trek Diagnostic Systems, Cleveland, OH, USA) for 4 h in the dark prior to the measurement of fluorescence, as described previously [30].

### Western Blotting

Cells were washed twice with TBS (10 mM Tris-HCl, pH 7.5, and 150 mM NaCl), lysed in the cell lysis buffer containing 10 mM sodium molybdate, 10 mM sodium fluoride, and 1 mM sodium orthovanadate, sonicated, and centrifuged at 20,000 × g for 10 min. The supernatant was subjected to SDS-PAGE, followed by Western blotting with rabbit monoclonal anti-phospho-AMPK (Thr172) (1/3000, #2535; Cell Signaling Technology, Beverly, MA, USA), monoclonal anti-AMPK (1/3000, #2532; Cell Signaling Technology), rabbit polyclonal anti-phospho-CREB (1/3000, Signalway antibody, College Park, MD, USA), and rabbit monoclonal anti-CREB (1/3000, #9197; Cell Signaling Technology). After incubation with horseradish peroxidase-conjugated secondary antibodies (Bio-Rad, Hercules, CA, USA), the immunoreactive bands were developed using Immobilon Western substrate (Millipore, Bedford, MA, USA) and read on LAS4000 system (GE Healthcare, Piscataway, NJ, USA). The band intensities were quantified using Image J (ver. 1.44p, National Institutes of Health, Bethesda, MD, USA).

### Real-time PCR

Total RNA was extracted from the cells using Sepasol RNA I Super G (Nacalai Tesque). After DNase I-treatment, cDNA was synthesized from total RNA using ReverTra Ace (TOYOBO, Osaka, Japan) and dT20 primer. Quantitative real-time PCR was performed using SYBR Premix Ex Taq II (TAKARA Bio, Shiga, Japan) with the following primers: C/EBPβ, forward: 5′-GCAAGAGCCGCGACAAG-3′ and reverse: 5′-GGCTCGGGCAGCTGCTT-3′; glyceraldehyde-3-phosphate dehydrogenase (GAPDH), forward: 5′- AAAATGGTGAAGGTCGGTGT-3′ and reverse: 5′-TTTGATGTTAGTGGGGTCTC-3′. The Ct values were transformed into relative expression levels using the standard curve method.

### Reporter assay

3T3-L1 cells grown on a 48-well plate to confluence were transfected with 0.2 μg of p4xCRE-TATA-Luc [30] and 0.1 μg of pGL4.74[hRluc/TK] using 0.75 μL of HilyMax reagent (Dojindo, Kumamoto, Japan) for 48 h. On day 0, the medium was exchanged to normal culture medium or fresh differentiation medium and the cells were incubated in the presence of 20 μM mogrol or 1 mM AICAR for 30 min. Then, cells were stimulated with insulin, DEX, and IBMX for 24 h. Luciferase activities were determined as described previously [31].

### Statistical analysis

Data were analyzed by analysis of variance followed by post-hoc Dunnett’s or Tukey’s tests using JMP software version 8.01 (SAS Institute, Cary, NC, USA). Data are expressed as means ± standard deviation (SD), and *p* values of less than 0.05 were regarded as statistically significant.

## Results

### Effects of mogrol and mogrosides on the lipid accumulation in 3T3-L1 cells

**Fig 1:**
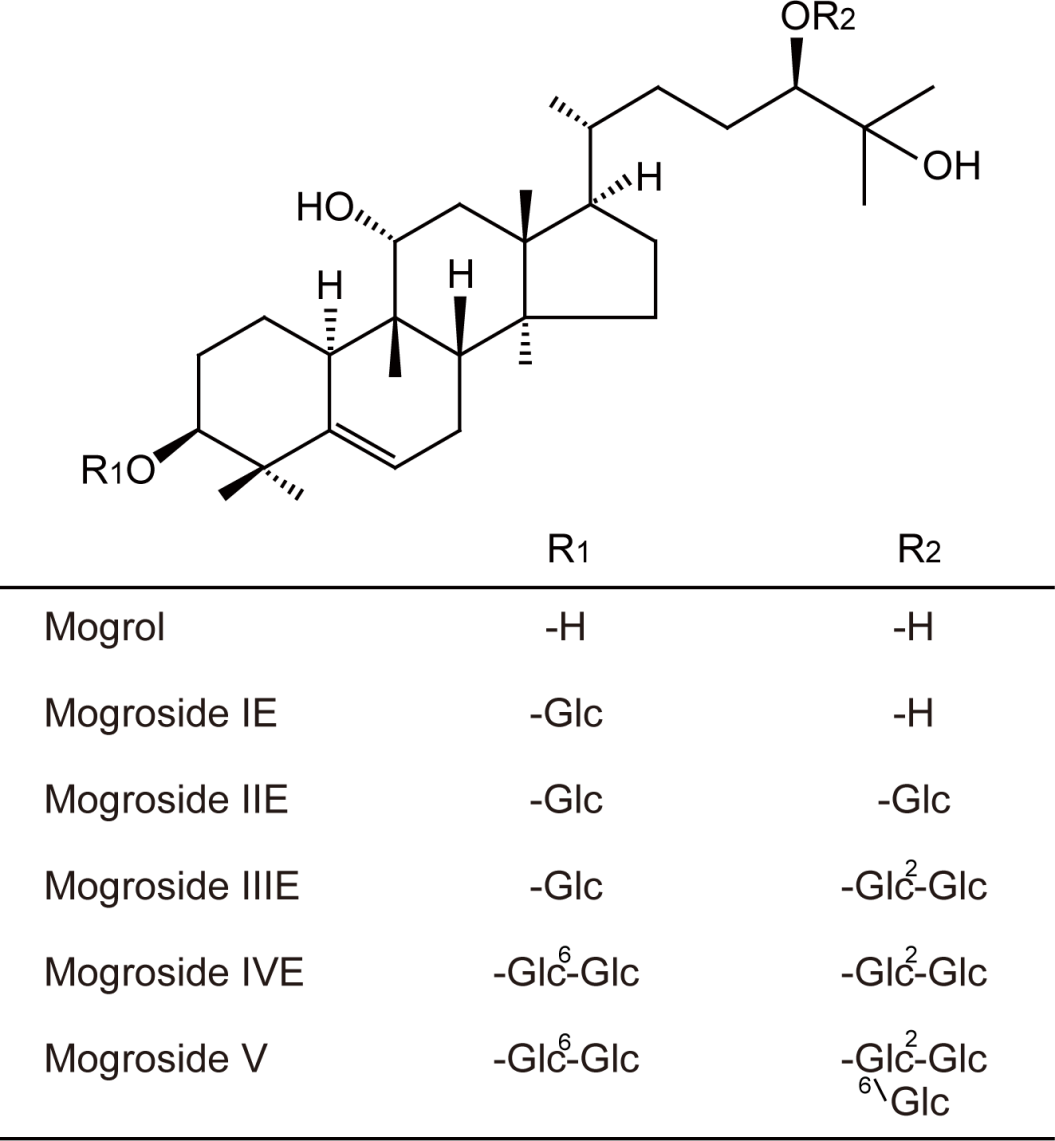
Chemical structures of mogrol and its glycosides isolated from *S*. *grosvenorii* fruit. Glc represents the β-D-glucopyranosyl group.

**Fig 2:**
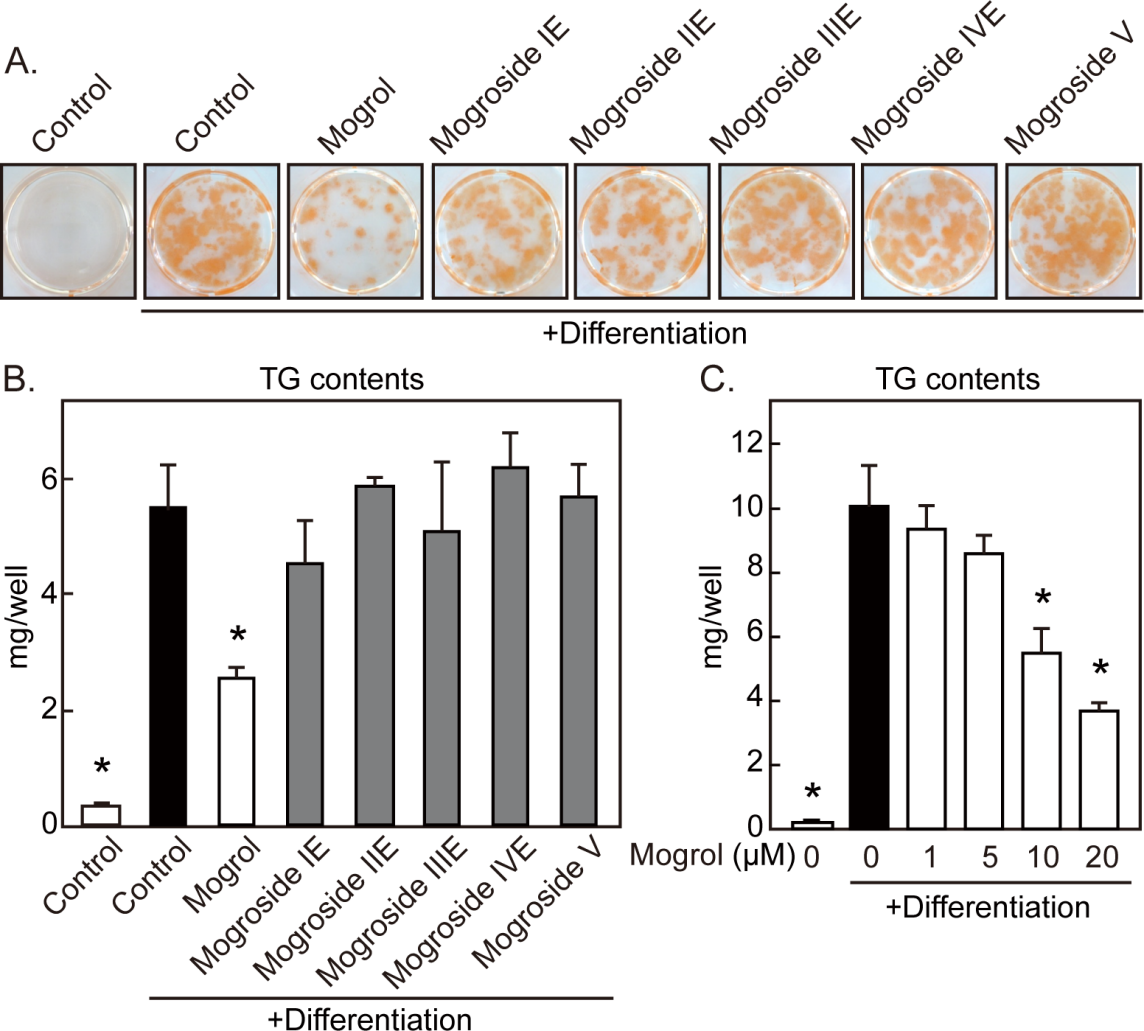
Effects of mogrol and its glycosides on lipid accumulation by 3T3-L1 cells during differentiation. 3T3-L1 cells were differentiated in the presence of mogrol (20 μM) or its glycosides (20 μM) for 8 days. (A) Representative images of Oil Red O staining derived from three independent experiments. (B,C) Mean ± SD cellular TG levels derived from a representative of independent experiments. Error bars indicate the SD of three replicates. *p < 0.05 for the comparison with cells differentiated in the absence of mogrol (solid black bar), one way ANOVA and Dunnett’s post-hoc testing.

The structures of mogrol, mogroside IE, mogroside IIE, mogroside IIIE, mogroside IVE, and mogroside V are presented in Fig 1. We examined the effects of mogrol and its glycosides at 20 μM on the differentiation of 3T3-L1 cells to adipocytes. Cell differentiation was evaluated by determining lipid accumulation using Oil Red O at day 8. The pigment-positive area was increased by differentiation stimuli (*i*.*e*., insulin, DEX, and IBMX) and was markedly decreased by mogrol (Fig 2A). Mogrol, but not mogrosides, significantly repressed the increase in cellular TG levels induced by differentiation stimuli (Fig 2B). Although mogroside IE tend to decrease TG levels, this effect did not reach statistical significance. Mogrol suppressed TG accumulation at micromolar levels, with a statistically significant suppression observed above 10 μM (Fig 2C).

### Effect of Mogrol on the Viability of 3T3-L1 Preadipocytes

**Fig 3:**
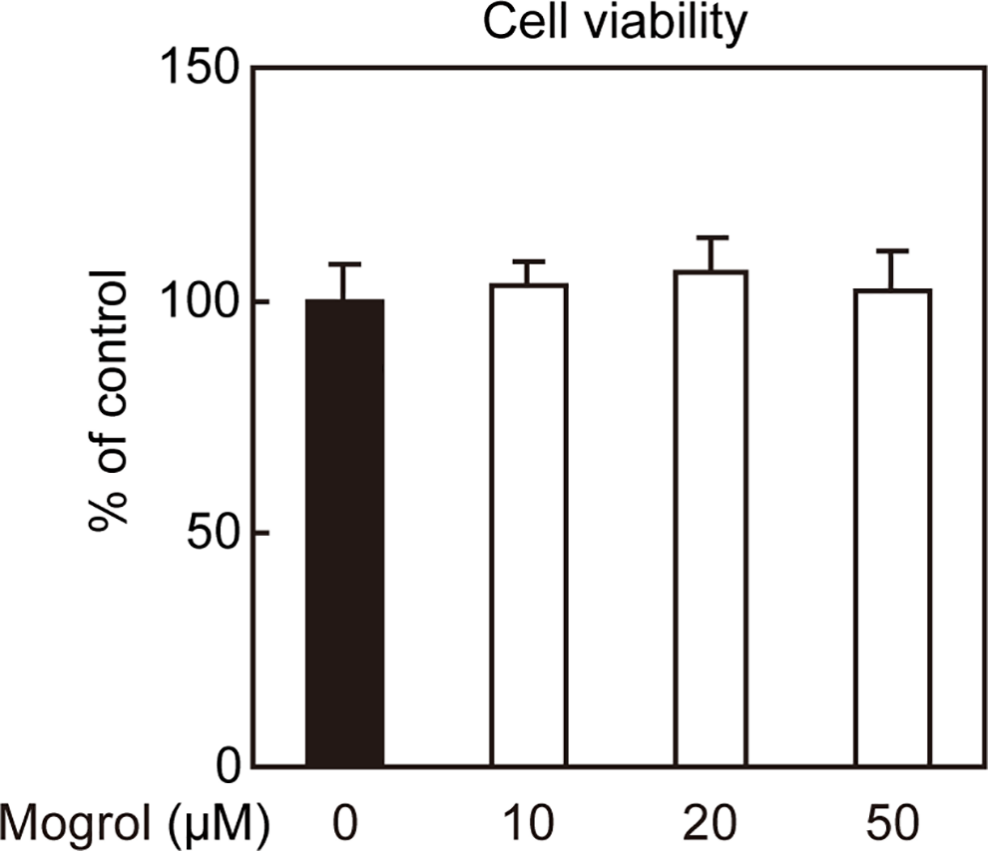
Effects of mogrol on the cell viability of 3T3-L1 preadipocytes. 3T3-L1 cells were incubated in the presence the indicated concentrations of mogrol for 8 days. Medium with mogrol was exchanged every two days. After incubation with 5% AlamarBlue dye for 4 h, the cell viabilities were determined. Data represent the mean ± SD of a representative of three independent experiments with four replicates. No statistically significant differences were observed for the comparison with cells in the absence of mogrol (solid black bar) using one way ANOVA with Dunnett’s post-hoc test.

We subsequently assessed whether the suppression of lipid accumulation by mogrol was due to an effect on cell viability. Mogrol did not affect the viability of 3T3-L1 preadipocytes at a concentration of up to 50 μM (Fig 3). These results suggested that mogrol suppressed adipogenesis in 3T3-L1 cells at concentrations that did not affect cell viability.

### Evaluation of Differentiation Stages Influenced by Mogrol

**Fig 4:**
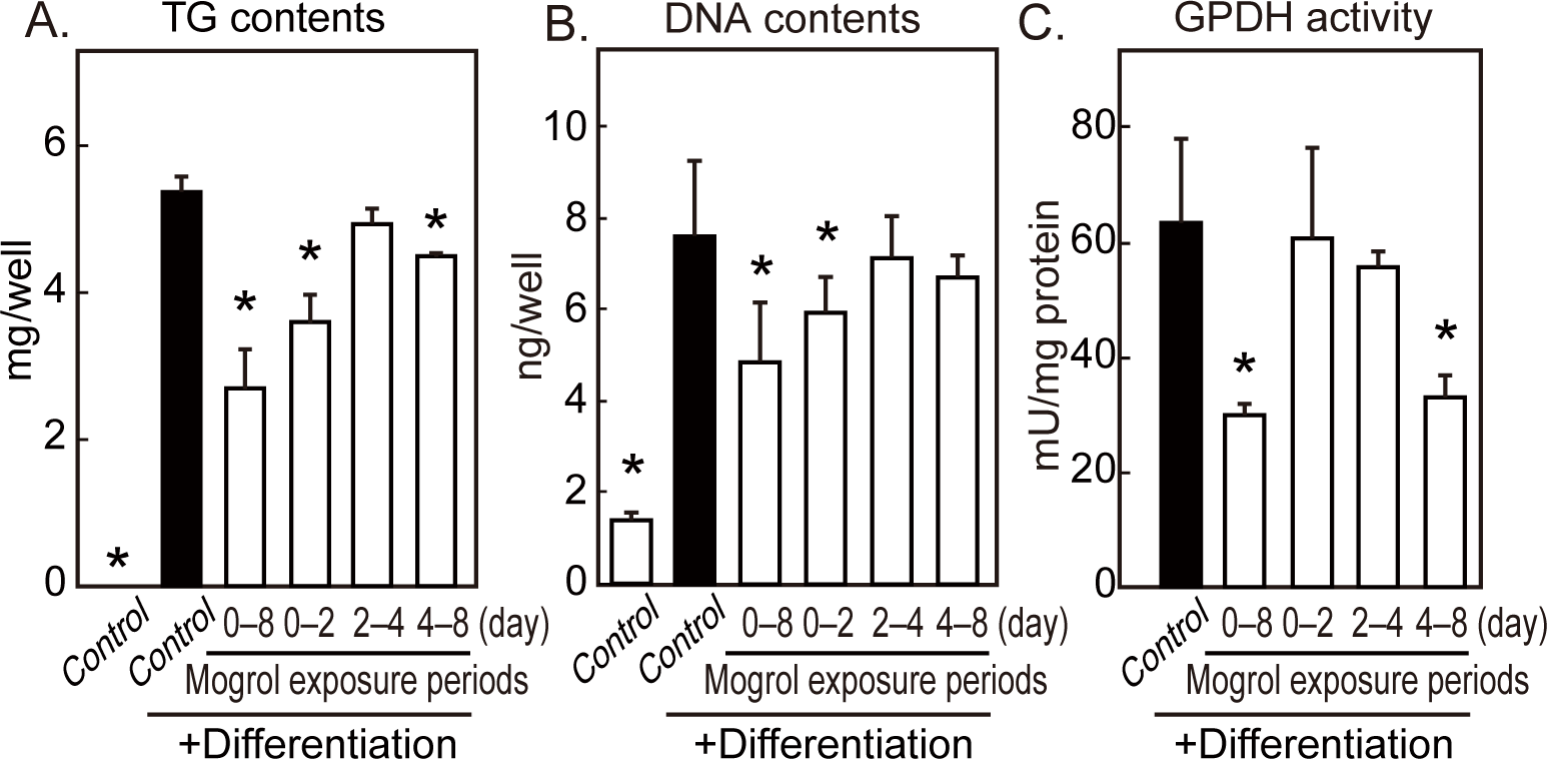
Effects of mogrol during the different stages of 3T3-L1 differentiation. 3T3-L1 cells were differentiated for 8 days and 20 μM mogrol was only added at the indicated stages of cell differentiation. The amounts of (A) triglyceride (TG), (B) DNA, and (C) glycerol-phosphate dehydrogenase (GPDH) activity were determined. Data represent the mean ± SD of a representative result of three independent experiments. Error bars indicate the SD of three (A, C) or six (B) replicates. *p < 0.05 for the comparison with cells differentiated in the absence of mogrol (solid black bar), one way ANOVA with Dunnett’s post-hoc test.

To examine the stage at which mogrol inhibited differentiation, 3T3-L1 cells were exposed to mogrol during specific differentiation periods. Mogrol suppressed the cellular TG level when presented at the early (days 0–2) and late (days 4–8) stages of differentiation, but not when presented in the middle stage of differentiation (days 2–4) (Fig 4A). The suppressive effect of mogrol was stronger in the early stage than in the late stage. In the early stage of 3T3-L1 adipogenesis, cell division (*i*.*e*., clonal expansion) is induced by differentiation stimuli. The 3T3-L1 cellular DNA levels (per culture dish) were significantly suppressed when the cells were exposed to differentiation stimuli in the presence of mogrol (Fig 4B). This effect was only observed when these cells were incubated with mogrol at the early stage of differentiation. On the other hand, GPDH activity was decreased in cells exposed to mogrol at the late stage of differentiation (Fig 4C). These results indicated that mogrol suppressed adipogenesis through at least two different mechanisms.

### Effects of Mogrol on AMPK phosphorylation

**Fig 5:**
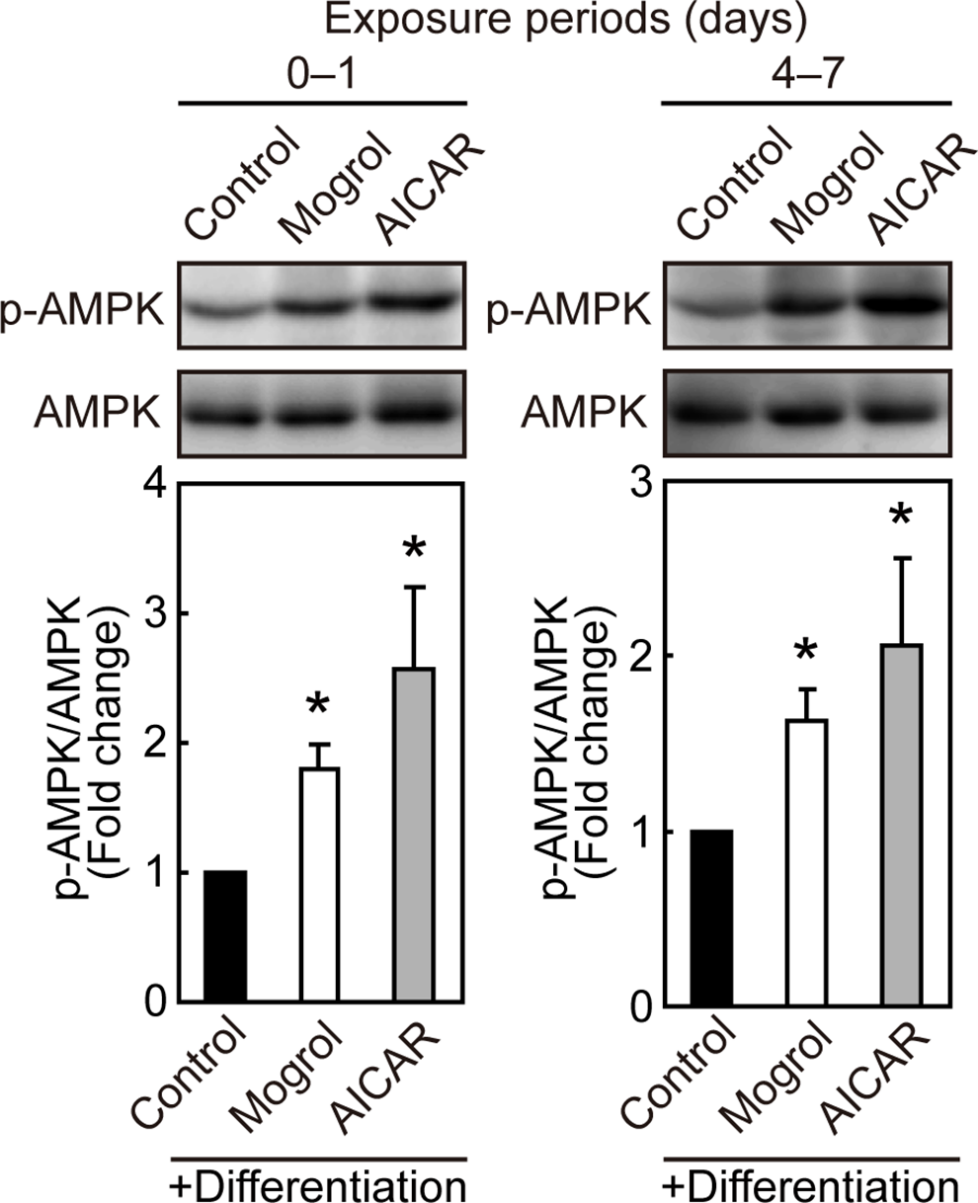
Effects of mogrol on the phosphorylation of AMP-activated protein kinase (AMPK). 3T3-L1 cells were differentiated in the presence of mogrol (20 μM) from days 0–1 or from days 4–7, with cell lysates were prepared on days 1 and 7, respectively. The cell lysates were analyzed by Western blotting with anti-phosphorylated-AMPK (p-AMPK) or anti-AMPK antibodies. Data are representative of four independent experiments with similar results. The band intensity of phosphorylated AMPK was expressed relative to the band intensity of total AMPK. Error bars indicate the SD (n = 4); *p < 0.05 for the comparison with cells differentiated in the absence of mogrol (solid black bar), one way ANOVA with Dunnett’s post-hoc test.

We examined the effects of mogrol on the activation of AMPK. 3T3-L1 cells were incubated with mogrol from days 0–1 (24 h) or days 4–7. In the latter case, the medium was exchanged with fresh medium containing fresh mogrol on day 6 and then incubated for an additional 24 h. Cell lysates were subjected to SDS-PAGE, followed by Western blotting. Mogrol and AICAR (a positive control for AMPK activation) increased AMPK phosphorylation on both days 1 and 7 (Fig 5).

### Determination of C/EBPβ Levels and PKA Signaling in Initial-Stage Adipogenesis

**Fig 6:**
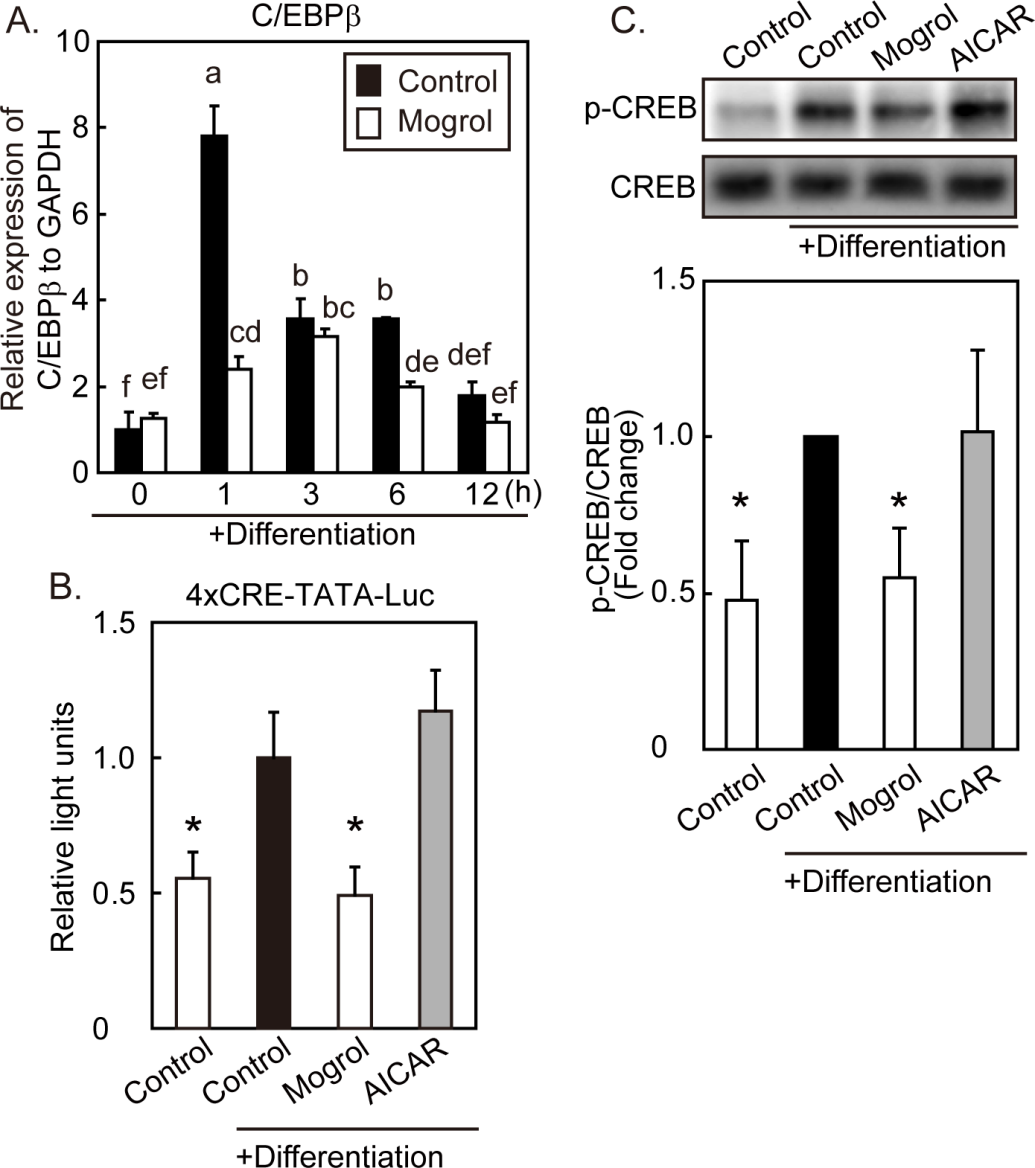
Effects of mogrol on CCAAT/enhancer-binding protein β (C/EBPβ) expression and cAMP response element (CRE)-mediated transcription. (A) On day 0, 3T3-L1 cells were pretreated with 20 μM mogrol for 30 min, and cells were stimulated by insulin, dexamethasone (DEX), and 3-isobutyl-1-methylxanthine (IBMX) for the indicated time-periods. Total RNA was extracted from the cells and the expression of C/EBPβ relative to that of glyceraldehyde-3-phosphate dehydrogenase (GAPDH) was analyzed by real-time PCR. Different letters mean statistically significant differences, determined by one way ANOVA with Tukey’s post-hoc test. (B) Two days before day 0, 3T3-L1 cells were transfected with p4xCRE-TATA-Luc and pGL4.74[hRluc/TK]. On day 0, the medium was exchanged to differentiation medium and the cells were stimulated with 0.5 mM IBMX for 24 h prior to the determination of luciferase activities. *p < 0.05 for the comparison with cells differentiated in the absence of mogrol (solid black bar), one way ANOVA with Dunnett’s post-hoc test. Data indicate the mean ± SD and are representative of three independent experiments with similar results. Error bars represents the SD of three (A) or four (B) replicates. (C) On day 0, cells that had been pretreated with mogrol or AICAR for 30 min in differentiation medium were stimulated by differentiation initiators for 5 min. The cell lysates were then analyzed for cAMP response element-binding protein (CREB) or phosphorylated CREB (p-CREB) by Western blotting. Photographs are representative of four independent experiments with similar results. The band intensity of phosphorylated CREB was expressed relative to the band intensity of total CREB. Error bars represent the SD of four independent experiments; *p < 0.05 for the comparison with cells differentiated in the absence of mogrol (solid black bar), one way ANOVA with Dunnett’s post-hoc test.

To examine the mechanism by which mogrol suppressed the number of 3T3-L1 cells (*i*.*e*., clonal expansion), we determined its effects on the expression of C/EBPβ. On day 0, 3T3-L1 cells were pretreated with mogrol for 30 min prior to the induction of differentiation. Quantitative real-time PCR analysis showed that the C/EBPβ mRNA levels induced by the differentiation stimuli were significantly suppressed by mogrol (Fig 6A). The expression of C/EBPβ and 3T3-L1 adipocyte differentiation are induced by IBMX, an activator of PKA signaling and cAMP response element (CRE)-mediated transcription [27, 32]. To examine the effects of mogrol on the transcriptional activity of CRE, we performed a luciferase reporter assay. 3T3-L1 cells were transfected with p4xCRE-TATA-Luc reporter vector and differentiation was induced. Differentiation stimuli increased luciferase activity; this increase was diminished by mogrol, but not by AICAR (Fig 6B). Similar to the effect on luciferase activity, phosphorylation of CREB was elevated after 5 min of differentiation stimuli, while mogrol (but not AICAR) suppressed the levels of phosphorylated CREB (Fig 6C). These results indicated that mogrol repressed the activation of CREB and CRE-mediated transcription at the initial phase of differentiation in 3T3-L1 cells, independent of its activation of AMPK signaling.

## Discussion

Mogrosides are the major active ingredients of *S*. *grosvenorii* and are used as a sugar substitute. Mogrol is an aglycone of mogroside, and is the major circulating form in the blood after the removal of the glucose residues associated with mogrosides in the gut [17]. In this study, we demonstrated that mogrol, but not its glycosides (*i*.*e*., mogroside I–V), suppressed the differentiation of 3T3-L1 preadipocytes into adipocytes. Moreover, our results indicated that the suppressive effects of mogrol were due to the repression of CREB activity and the activation of AMPK signaling in 3T3-L1 cells.

Mogrol reduced TG accumulation at early (days 0–2) and late (days 4–8) stages, but not at middle (days 2–4) stage. In both the early and late stages, mogrol increased the levels of phosphorylated AMPK. In both stages, activation of AMPK leads to suppression of TG accumulation [13]. In the early stage of differentiation, activation of AMPK by AICAR enhances WNT/β-catenin signaling and suppresses clonal expansion [12–14]. These mechanisms seem to reduce subsequent TG accumulation. Activation of AMPK inactivates acetyl-CoA carboxylase, which leads to a reduction of lipogenesis and an increase lipid β-oxidation [33]. Since acetyl-CoA carboxylase is increased and lipogenesis is accelerated during late-stage differentiation [5, 6], these mechanisms are considered to be responsible for the decrease in TG accumulation at this stage. Mogrol also suppressed GPDH activity at the late stage of differentiation. GPDH activity is a TG accumulation-independent marker of adipocyte differentiation [9] and AMPK activation by mogrol may therefore reduce late-stage terminal differentiation. This may result from a repression of PPARγ activity, which is essential for adipocyte differentiation [4, 5] and is suppressed by AMPK via phosphorylation of p300 [34, 35].

Mogrol suppressed the induction of C/EBPβ during the initiation of 3T3-L1 differentiation. On the other hand, AICAR suppresses 3T3-L1 differentiation without reducing the induction of C/EBPβ during the early stage [12, 13]. Unlike AICAR, mogrol suppressed the induction of CRE-mediated transcription and CREB phosphorylation. C/EBPβ acts as a master regulator for the initiation of adipocyte differentiation [4, 5] and clonal expansion [36], inducing the expression of key downstream transcription factors involved in adipocyte differentiation (*e*.*g*., C/EBPα and peroxisome proliferator-activated receptor γ) [4, 36]. The expression of C/EBPβ is mainly regulated by IBMX (*i*.*e*, CRE-mediated transcription) [27, 32] and the suppressive effects of mogrol on CREB activation and CRE-mediated transcription are therefore considered to be important for inhibition of 3T3-L1 cell differentiation.

In 3T3-L1 cells, mogrol suppressed TG accumulation at micromolar levels, with a statistically significant suppression observed at > 10 μM (Fig 2C). A previous study showed that the portal blood mogrol concentration was 0.36 μM in rats 2 h after a single oral administration of 65.5 μmol (84 mg) mogroside V [17]. In addition, our unpublished results indicated that 1 h after a single oral administration of 5 μmol (2.4 mg) mogrol, the rat portal blood mogrol concentration was > 4 μM. Taken together, it seems possible that the concentrations of mogrol can reach the effective dose *in vivo* when ingested repeatedly.

AMPK signaling is influenced by polyphenols including berberine [37], genistein [38], epigallocatechin gallate [38], capsaicin [38], apigenin [39], nobiletin [40], resveratrol [41], and quercetin [42], all of which suppress lipid accumulation in 3T3-L1 cells. In this study, we showed that mogrol also phosphorylated AMPK in 3T3-L1 cells, similar to its role in hepatocytes [25]. Forskolin (an activator of adenylate cyclase) and cAMP analogs activate AMPK in 3T3-L1 cells during late-stage differentiation [11]. Therefore, mogrol is considered to activate AMPK signaling via another mechanism that does not involve inhibition of PKA signaling. Mogrol exerted similar effects as berberine on 3T3-L1 differentiation. Like mogrol, berberine suppressed TG accumulation at the early and late, but not the middle, stage of differentiation [43]. In addition, mogrol and berberine suppressed the phosphorylation of CREB and the induction of C/EBPβ at the initial differentiation stage and activated AMPK at both early and late differentiation stages [43]. On the other hand, although genistein inhibits the DNA binding of C/EBPβ in 3T3-L1 cell differentiation without affecting its total and nuclear protein levels [44], it remains unclear whether genistein affects the activation of CREB. Many food constituents have been reported to activate AMPK signaling, while their effects on CREB activation remain largely unclear. However, our results suggest that the phosphorylation of CREB is a target for the suppression of adipogenesis by some functional foods.

## Conclusions

In the present study, mogrol suppressed lipid accumulation by 3T3-L1 adipocytes, presumably by repressing CREB phosphorylation and CRE-mediated transcription in the initial stage of differentiation, and by activating AMPK signaling at both the early and late stages.
